# Sequential in-office vitreous aspirates demonstrate vitreous matrix metalloproteinase 9 levels correlate with the amount of subretinal fluid in eyes with wet age-related macular degeneration

**Published:** 2012-06-20

**Authors:** Stephanie M. Ecker, Scott M. Pfahler, Joshua C. Hines, Ann S. Lovelace, Bert M. Glaser

**Affiliations:** 1 The National Retina Institute, Department of Ocular Proteomics, Towson MD; 2 Ocular Proteomics LLC, Towson MD

## Abstract

**Purpose:**

To evaluate levels of 37 native pathway proteins of the vitreous proteome from a subset of wet age-related macular degeneration (AMD) patients with and without subretinal fluid (SRF).

**Methods:**

A total of 62 consecutive samples were aspirated from 12 patients with AMD, six who had SRF at baseline, and six who did not have SRF at any point during the study. Vitreous levels of the 37 native pathway proteins were analyzed in these patients using reverse phase protein microarray technology. At each visit, at which the 62 samples were taken, SRF and central retinal thickness were measured. These values were then compared to the relative intensity level of the 37 proteins screened.

**Results:**

In the subset of AMD patients with SRF, the average matrix metalloproteinase 9 (MMP-9), interleukin (IL)-12, Abelson murine leukemia viral oncogene homolog 1 (cABL) Thr735, heme oxygenase-1, Musashi, platelet-derived growth factor receptor beta Tyr751 (PDGFRβ), IL-8, and BCL-2 associated death promoter (BAD) Ser112 levels in the vitreous were found to be significantly different with a 21%–82% increase in expression compared to those without SRF (p<0.0001). Within the SRF group, there was a positive correlation between the vitreous MMP-9 levels and the SRF level. MMP-9 levels in the vitreous proteome varied with the level of SRF but not retinal edema. Compared to patients without SRF, the patients with initial SRF had persistent or progressive disease.

**Conclusions:**

This is the first prospective case series sequentially monitoring the vitreous proteome in patients with wet AMD. The results suggest that MMP-9 is a proteomic biomarker of SRF accumulation, separate from macular edema.

## Introduction

Exudative or wet age-related macular degeneration (AMD) is a complex disease. The heterogeneity of disease expression and the differences in patient response to antiangiogenic bevacizumab or ranibizumab treatment suggests that vascular endothelial growth factor (VEGF) is unlikely to be the only protein of interest in this disease. In fact, studies have shown proteins such as VEGFR2 Y1175 and Y951 [1], transforming growth factor-beta 1 and monocyte chemotactic protein-1 [2], and C-reactive protein and interleukin-6 (IL-6) [3], may play a role in determining the clinical course of AMD. These studies imply that the determining factors of AMD may vary from patient to patient and investigating the vitreous proteome for a wide range of potential biomarkers will be integral in defining the biologic pathways that are important in the disease.

Previous work has demonstrated the safety and efficacy of taking small vitreous aspirations in an in-office setting for use with reverse phase protein microarray (RPPM) technology for analyzing the samples against a wide array of pathway proteins [1,4]. Here, a pilot study was designed to measure the vitreous levels of 37 activated or native pathway proteins representing angiogenesis, apoptosis, inflammation, and hypoxia/oxidative stress pathways against quantifiable clinical findings in AMD, including central retinal thickness (CRT) and subretinal fluid (SRF). SRF and CRT levels were chosen for study because significant differences among patients are regularly observed. Multiple sequential vitreous aspirates (3–9) were taken from each study patient and analyzed to understand if a correlation exists with levels of SRF and CRT. The results of this study demonstrate that matrix metalloproteinase 9 (MMP-9) is significantly associated with the development of SRF in AMD patients. Since MMP-9 expression correlates with levels of SRF in retinal detachment [5,6], this analysis may lead to a unifying understanding of why SRF accumulates.

## Methods

This prospective series included 62 consecutive samples from patients with exudative AMD who had undergone in-office diagnostic vitreous sampling before anti-VEGF agent or corticosteroid was intravitreally injected at multiple time points [4]. Twelve patients with AMD were enrolled: six with SRF and six with retinal thickening but no SRF (see Table 1).

**Table 1:** Patient demographics.

**Number of patients**	12
Average age	78
Age range	66–90
**Sex**	
Male	4
Female	8
Total vitreous samples	62
Avg per patient	5.1
Range	(3–9)
Avg follow-up (days)	165
Range (days)	(70–279)

This research study and vitreous aspiration procedure has institutional review board approval (Western Institutional Review Board, Olympia, WA) and is Health Insurance Portability and Accountability Act–compliant with written informed consent received from all study patients. Patients were selected for this study based on a set of inclusion and exclusion criteria. The inclusion criteria included the following: 1) Patients with exudative AMD that required intravitreal medication injection (anti-VEGF or corticosteroid) and 2) who consented to undergo vitreous sampling. The exclusion criteria were as follows: 1) Patients unwilling or unable to consent to study or follow-up, 2) ophthalmic surgery within the last three months, 3) active intraocular inflammation, and 4) recent cerebral vascular accident or myocardial infarction.

At each visit, study patients received a standard examination that included a detailed medical history, ETDRS best-corrected visual acuity, intraocular pressure, and a dilated retinal exam. All patients had intravenous fluorescein angiography, indocyanine green angiography, and optical coherence tomography (Spectralis HRA+OCT, Version 5.3.3; Heidelberg Engineering, Heidelberg, Germany), at baseline and every subsequent visit. If SRF or retinal thickening was present, patients underwent diagnostic vitreous sampling (DVS) followed by an intravitreal injection of an anti-VEGF agent or corticosteroid agent. Patients with SRF were assigned to Group 1, while patients with retinal thickening without SRF were labeled Group 2 (see Table 2 and Table 3). Patients were followed at monthly intervals.

**Table 2:** Group 1 (subretinal fluid) demographics.

**Patient**	**Age**	**Initial VA**	**Final VA**	**Study vitreous sample/injections**
1	85	20/63	20/40	4
2	70	20/50	20/20	7
3	66	20/100	20/100	7
4*	90	20/50	20/80	7
5*	63	20/100	20/63	6
6*	73	20/63	20/32	8

* Patient 4 and 5 had intravitreal triamcinolone, at visit 8 and 6 respectively. Patient 6 was switched to ranibizumab on visits 6–7. All other injections were bevacizumab 0.05 ml/1.25 mg.

**Table 3:** Group 2 (no subretinal fluid) demographics.

**Patient**	**Age**	**Initial VA**	**Final VA**	**Study vitreous samples/injections**
7	80	20/160	20/100	3
8	88	20/100	20/80	4
9	86	20/200	20/200	4
10	74	20/80	20/63	3
11	82	20/800	20/640	3
12	82	20/40	20/40	4

Spectral domain OCT images were obtained using the standard volume scan algorithm that acquires 19 horizontal B-scans of the macula (7 µm resolution). All subsequent scans were registered to the initial scan (reference scan) allowing accurate point-to-point measurements on follow-up. The caliper tool allowed the SRF height to be measured (distance between the hyper-reflective band corresponding to the inner aspect of the retinal pigment epithelium [RPE] to the hyper-reflective band corresponding to the photoreceptor inner segment junction and outer segments) at exact locations on follow-up. In Group 1, the point of the greatest linear height of SRF was measured and followed at each time interval. If SRF resolved and a new area of fluid developed, the new location was followed (two patients). In both groups, a linear calculation of retinal thickness was performed at the site of SRF measurement or at the site of greatest thickening for patients without sub-retinal fluid. Spectralis HRA+OCT software’s automated retinal thickness algorithm was used for thickness measurements. Each measurement was inspected to ensure that the appropriate retinal boundaries were marked (see Appendix 1).

DVS [4] was performed by taking a small vitreous aspirate before an injection of bevacizumab 0.05 ml/1.25 mg (Avastin; Genentech, South San Francisco, CA), ranibizumab 0.05 ml/1.25 mg (Lucentis; Genentech), or triamcinolone acetonide 0.10 ml/4 mg (Kenalog; Bristol-Meyer-Squibb, New York, NY). Vitreous samples were obtained by using a standard technique on all patients as follows: Topical anesthesia was achieved by instilling topical lidocaine gel 2% (Xylocaine; AstraZeneca LP, Wilmington, DE) followed by placement of a soaked cotton-tip applicator (lidocaine 4% solution; Roxane Laboratories, Columbus, OH) in the inferior temporal quadrant. Using sterile gloves, a sterile lid speculum was placed followed by a drop of Betadine 5% (Alcon, Fort Worth, TX). Immediately following, a 25 gauge needle (Terumo Needle; Terumo Medical Corporation, Elkton, MD) was placed via the pars plana approach into the mid-vitreous cavity, and 0.05 to 0.10 ml of vitreous fluid was aspirated into a 1 ml syringe (PrecisionGlide; Becton Dickinson, Franklin Lakes, NJ). After the needle was removed, a 30 gauge needle (PrecisionGlide; Becton Dickinson) was introduced into the same location and the injection performed. Intraocular pressure was measured only if a vitreous sample was not obtained (“dry sample”). After injection, the patient was discharged with post-injection warnings and instructions to use a fourth-generation fluoroquinolone antibiotic drop (moxifloxacin or gatifloxacin) four times a day for 5 days. Patients were educated regarding the warning signs that require immediate evaluation, including pain, decreased vision, loss of peripheral vision, redness, or sensitivity to light. Patients were followed at intervals of 1 month. Safety and adverse events were documented at all follow-up visits using a standardized history questionnaire completed by the examining physician.

**Figure 1:**
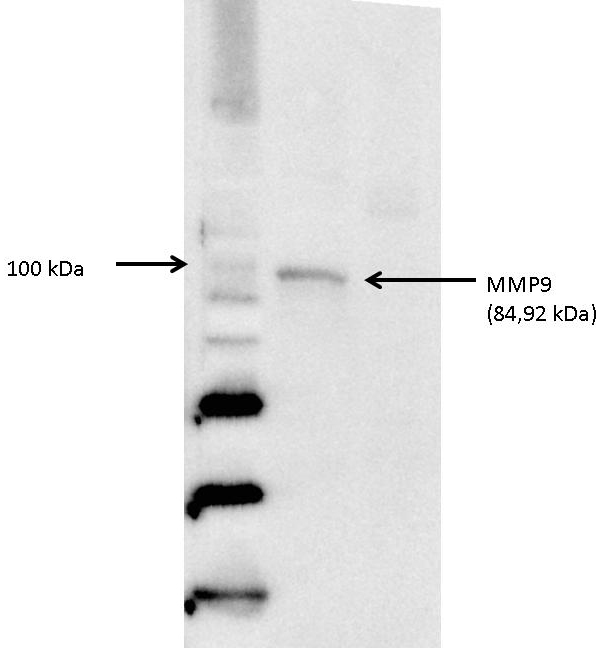
Validation of matrix metalloproteinase 9 (MMP-9) antibody (Cell Signaling Cat# 3852, Lot#2) via western blot. The molecular weight of MMP-9 is 84, 92 kDa. Lanes 1–3 go Left to Right. Lane 1: is a Magic Mark Western Standard (Cat # LC5602, Life Technologies) molecular weights ranging from 20 to 220 kDa. Lane 2, shows the validation of the MMP-9 antibody, using human endothelial cell lysates (BD Cat# 611450, Lot: 86722). Lane 3 shows is a negative control loaded with BSA (BSA; Thermo Fisher Product # 23209).

Vitreous samples were analyzed with RPPM technology as follows: 37 antibodies to proteins known to be important in angiogenesis, proliferation, inflammation, and oxidative stress pathways were selected and validated in-house with the western blot technique (see Appendix 2). The validated antibody blot for MMP-9 can be seen in Figure 1. The total protein concentration of each vitreous sample was measured spectrophotometrically with the Bradford method. Each sample was diluted in extraction buffer (45% T-Per; Pierce, Indianapolis, IN), 10% T-Cep (Fisher Scientific, Fair Lawn, NJ), and 45% 2X SDS Tris-glycine loading buffer (Invitrogen, Carlsbad, CA) to equalize the total protein loaded to 0.1 μg/ml. The samples were printed on glass nitrocellulose coated array slides (FAST slides, Whatman, Florham Park, NJ) using an Aushon 2470 arrayer (Aushon Biosystems, Burlington, MA) with 350 μm pins. Samples were set up to be printed in duplicate dilution curves representing the neat, 1:2, 1:4, and 1:8 dilutions. For quality control purposes, control cell lysates prepared from A431± EGF, HeLa ±Pervanadate, human endothelial cells (Becton Dickinson, Franklin Lakes, NJ), and BSA were printed on each array in the same manner, including an eight-point dilution curve from neat to 1:128. The arrays were stored with desiccant (Drierite; W.A. Hammond, Xenia, OH) at −20 °C before immunostaining [1]. Immunostaining was done following the same protocol as described [7] with monoclonal or polyclonal primary antibodies (Appendix 2) and biotinylated secondary antibodies using the Dako autostainer and CSA staining kit (Dako, Fort Collins, CO).

Spot intensity analysis was done in the same manner as [7] using ImageQuant (v5.2; GE Healthcare, Piscataway, NJ) to capture the intensity of every spot on the array. The raw spot intensity data were then entered into Microsoft Excel (Microsoft, Redmond, WA) for normalization, which includes ensuring the duplicate spots are within 10% coefficient of variation (CV) of each other, averaging the duplicate spots and performing a curve fitting analysis to determine the dilution point with the best signal to noise ratio. This normalization process leads to a single protein expression value for each sample on the array that represents the relative intensity (RI), which can be referenced to all other samples on the array and between arrays [7].

Statistical analyses were performed using SAS Version 9.1.3 (SAS Institute, Cary, NC) and Prism 5.0 (GraphPad, La Jolla, CA). A *t* test and repeated measures ANOVA were used to compare the vitreous protein levels between the two groups of patients with AMD, and to ensure that patient clustering did not affect the outcome of the *t* test, a regression model using group clustering was applied. Receiver operator characteristic (ROC) analyses were also calculated to understand if the analytes could significantly determine differences between the SRF and no SRF groups. The correlation of each vitreous analyte to the subsequent SRF level was done via Pearson’s correlation coefficients analysis.

## Results

This study analyzed 62 sequential samples aspirated from 12 patients with exudative AMD being treated with intravitreal therapies. Group 1 was composed of six patients with a total of 41 samples from patients who had baseline SRF, while Group 2 was also composed of six patients with a total of 21 samples from patients who had no SRF throughout the entire treatment (Appendix 1). The protein expression levels were measured for all samples in Groups 1 and 2. Of the 37 proteins screened, eight were significantly different between Group 1 and 2 using both a *t* test and a repeated measures ANOVA (see Table 4 and Table 5).

**Table 4:** *t*-test results showing the proteins with significantly different levels between Group 1 and Group 2.

**Protein**	**p-value**	**Percent difference**
MMP-9	p<0.0001	82.1% higher in Group 1
IL-12	p=0.0018	33.5% higher in Group1
cABL T735	p=0.0040	30.6% higher in Group 1
Heme Oxygenase-1	p=0.0064	26.2% higher in Group 1
Musashi	p=0.0175	45.9% higher in Group 1
PDGFRβ Y751	p=0.0238	21.3% higher in Group 1
IL-8	p=0.0355	23.0% higher in Group 1
BAD S112	p=0.0478	23.4% higher in Group 1

The significant results from *t*-test of each protein screened for group1 (patients with subretibnal fluid [SRF]) versus group 2 (patients with no SRF). Eight proteins (listed below) had a significant elevation in expression level in the group 1 patients, with matrix metalloproteinase 9 (MMP-9) showing the most significant change.

**Table 5:** Repeated measures ANOVA results showing the proteins with significantly different levels between Group 1 and Group 2.

**Protein**	**p-value**
MMP-9	p<0.0001
IL-12	p=0.0007
cABL T735	p=0.0018
Heme Oxygenase-1	p=0.0318
Musashi	p=0.0127
PDGFRβ Y751	p=0.001
IL-8	p=0.0338
BAD S112	p=0.0084

The Results of a repeated measures ANOVA, showing that the same eight proteins as in Table 4, are significantly different when comparing group 1 and group 2 patients.

Vitreous levels of MMP-9 were the most significantly different between Group 1 and 2. The MMP-9 value for Group 1 (with SRF) had an average vitreous expression level of 1.267 RI, which is 82.05% higher than the average MMP-9 expression level of 0.2473 RI in Group 2 (no SRF; p≤0.0001). A regression model, which considered clustering that could occur for each patient, was applied to these data, and the results validate the significant difference between the mean MMP-9 values for Groups 1 and 2 (p=0.0005). As shown in Table 4, MMP-9 has the most significant difference between Groups 1 and 2, yet al.l significant proteins move higher when SRF is present.

**Figure 2:**
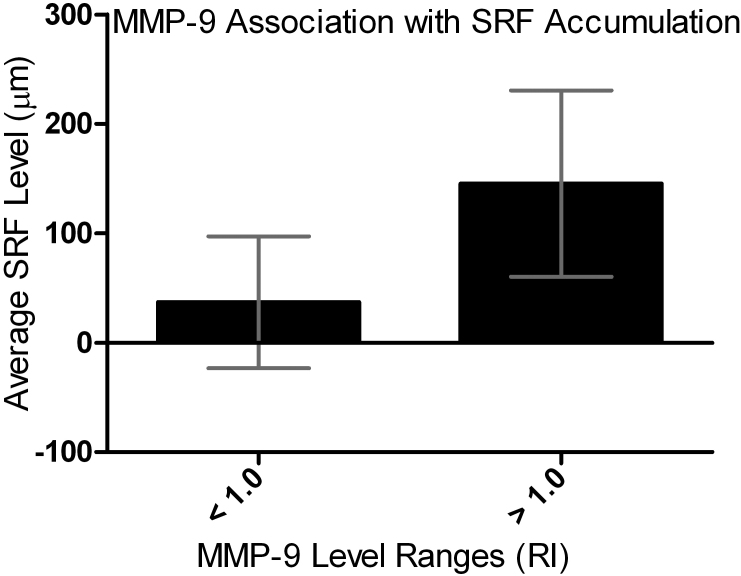
Matrix metalloproteinase 9 (MMP-9) expression in the vitreous is predictive of subretinal fluid (SRF) accumulation . Here it can be seen that when MMP-9 expression levels are below 1.0 patients generally have lower levels or no SRF accumulation, but when MMP-9 levels go above 1.0, patients have higher levels of SRF accumulation.

**Figure 3:**
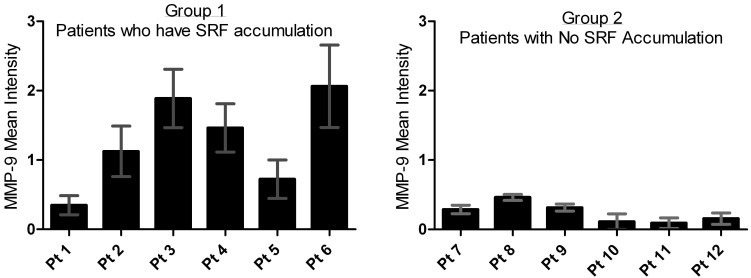
Matrix metalloproteinase 9 (MMP-9) expression differences between patients with SRF versus patients with no subretinal fluid (SRF). The MMP-9 mean expression level (±SD) is shown for each patient in this study. The Group 1 (Patients with SRF) graph shows that most patients have a mean MMP-9 expression level greater than 1.0. Whereas the Group 2 (Patients with NO SRF) graph shows that all patients have a mean MMP-9 expression level much lower than 1.0.

MMP-9 showed the most significant changes, so it was studied further to understand how the expression levels correspond to patient SRF levels. These MMP-9 results for Groups 1 and 2 were split into two expression level groups, and the mean SRF level for each group was calculated (Figure 2). When MMP-9 levels were below 1.0 RI, the average SRF was 36.92 µm, but when the MMP-9 level was above 1.0 RI, the average SRF level increased to 145.56 µm (p≤0.0001). To visualize trends in the data, a graphical representation (as shown in Figure 3) illustrates the mean vitreous MMP-9 levels for each patient in Groups 1 and 2. This shows that the patients who had no SRF had much lower MMP-9 expression, and further suggests an association between MMP-9 levels and SRF accumulation in patients with AMD.

**Figure 4:**
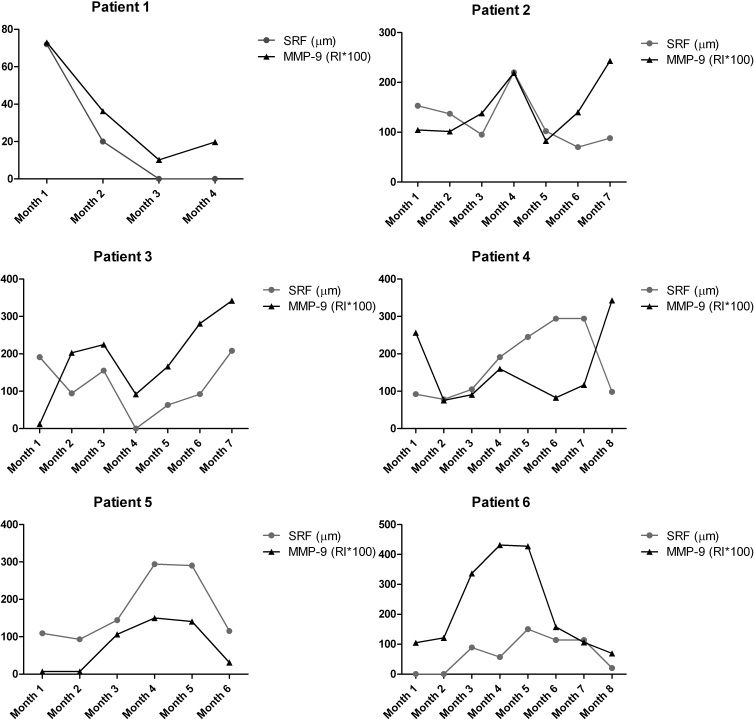
Matrix metalloproteinase 9 (MMP-9) tracks patient subretinal fluid (SRF) levels. The MMP-9 and SRF levels for patients 1–6 are plotted for each patient treatment visit. These graphs demonstrate how MMP-9 expression levels in the vitreous track the rise and fall of SRF in each patient over time.

**Figure 5:**
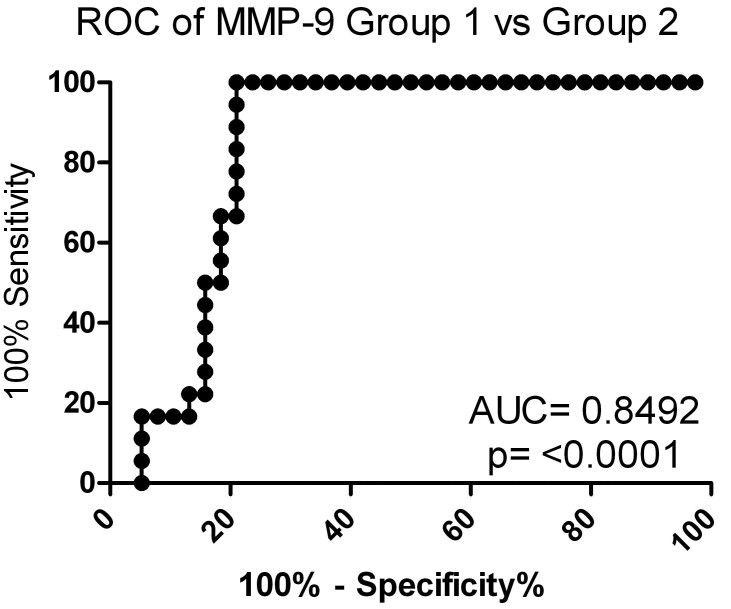
Sensitivity and specificity of matrix metalloproteinase 9 (MMP-9) association with subretinal fluid (SRF) accumulation. Receiver operator characteristic (ROC) curve analysis demonstrates MMP-9 to have a significant ability to distinguish patients who have SRF from patients who do not have SRF accumulation, with an area under curve (AUC) of 0.8492 and a p-value of <0.0001. This data could be used to determine a threshold level of MMP-9 that would be indicative of SRF accumulation, and possible worsening of AMD pathology.

Pearson’s correlation analyses were calculated to determine if the SRF levels from Group 1 correlate with their corresponding MMP-9 levels. The results show a positive correlation between SRF levels and vitreous MMP-9 levels for each Group 1 SRF patient, which ranged from 0.1540 to 0.9857. Figure 4 illustrates these findings, by showing how MMP-9 levels reflect and track SRF accumulation in each patient. The correlation between MMP-9 levels and SRF accumulation may suggest a possible prognostic ability for MMP-9 in patients with AMD, and other retinal diseases affected by SRF accumulation. To further explore this idea, a ROC analysis was completed to test the capability of MMP-9 to detect differences between Group 1 and Group 2 (Figure 5). This analysis revealed that MMP-9 specifically and sensitively distinguished the difference between Group 1 and 2 (area under the curve=0.843, p≤0.0001), further demonstrating the strong difference in MMP-9 between patients who have SRF and those who do not.

Each of the other seven significantly changed proteins between Group 1 and Group 2 were analyzed using Pearson’s correlation analysis to determine if the Group 1 patients, with SRF accumulation, showed a significant relationship to the expression levels. Unlike MMP-9, which had positive correlations with SRF levels in each patient, IL-12, cABL T735, heme oxygenase-1, Musashi, platelet-derived growth factor receptor beta (PDGFR beta) precursor Y751, IL-8, and BAD Ser112 had positive and negative correlations; therefore, we are unable to propose a connection between SRF and protein expression levels for these proteins (Table 6).

**Table 6:** Pearson’s correlation results for IL-12, BAD Ser 112, cABL Thr 735, heme oxygenase-1, IL-8, Musashi and PDGFRβ Y751.

**Patients**	**IL-12**	**c-Abl T7hr35**	**HemeOx-1**	**Musashi**	**PDGFRβ Y751**	**IL-8**	**Bad Ser112**
Patient 1	0.94868	−0.10541	−0.10541	0.948683	0.632456	0.948683	0.105409
Patient 2	0.71428	0.50000	0.714286	0.071429	0.142857	0.678571	0.178571
Patient 3	−0.21429	−0.53571	−0.60714	−0.10714	0.357143	−0.21429	−0.35714
Patient 4	0.25150	−0.35929	−0.32336	0.646718	0.850315	0.514979	0.083834
Patient 5	0.60000	0.485714	0.60000	0.657143	0.657143	0.60000	0.60000
Patient 6	−0.43377	−0.44582	−0.0482	−0.0482	−0.26508	−0.40967	−0.45786

The correlation coefficients are reported for each.

Retinal thickness was measured for each patient in this study, and the average thickness for Group 1 was 216 microns (range 170–309 µm) while Group 2 was 485 microns (range 297–779 µm). After intravitreal treatment (anti-VEGF or triamcinolone), the final average thickness for Group 1 was 264 microns (196–422 µm) versus 243 microns (120–318 µm) for Group 2. All patients except one in Group 2 had complete resolution of their initial edema. No strong correlations between vitreous MMP-9 levels and retinal thickening were observed.

## Discussion

Exudative AMD is a complex disorder with each patient having individual pathologic components occurring at different times and with different sequences. We are currently learning about genes that may be associated with AMD and still need to determine how to detect when these genes become active [8]. Vitreous proteomics can now be performed as an in-office procedure, and biomarkers can be measured over time so that disease progression may be predicted [1,4]. Vitreous proteomics provides a promising opportunity to map the activity of pathways active in each individual patient so that treatment can be optimized and a new approach to treatment can be developed. Our pilot study was designed to measure the vitreous levels of 37 native pathway proteins representing angiogenesis, apoptosis, inflammation, and hypoxia/oxidative stress pathways against quantifiable clinical findings in AMD including CRT and SRF. Multiple sequential vitreous aspirates (3–9) were taken from each study patient and analyzed to understand if a correlation exists with levels of SRF and CRT. The results suggest that MMP-9 is a potential biomarker in the development of SRF in patients with AMD. Though SRF accumulation can be seen via OCT, elucidating its biologic mechanisms will have much greater implications for future research. Further, this type of research could initiate predictive treatment capabilities within ocular medicine in the future.

MMPs are a complex family of enzymes capable of degrading the major components of the extracellular matrix (ECM) and are associated with processes involving ECM remodeling, development, angiogenesis, and inflammation [9]. MMP-9 is also known as gelatinase B due to its specificity for type IV collagen and its role in breaking down basement membranes [9]. There is evidence suggesting MMP-9 is associated with components of the Bruch’s membrane-RPE complex and is secreted by retinal pigment epithelial cells, and secretion of MMP-9 may result in defects in Bruch’s [10]. Such a defect in Bruch’s membrane may result from the overexpression of MMPs in the outer retinal complex and likely enables various events, possibly including the development of SRF and facilitating the cascade of choroidal neovascularization. Although the complete role of these factors has not yet been elucidated, they have been associated with basal laminar deposits [10], laser-induced choroidal neovascularization [11–13], and choroidal neovascular membranes associated with AMD [11,14]. MMPs have also been associated with RPE cells and the inter-photoreceptor matrix [10,15].

Recent reports have found that levels of MMP-9 in SRF and vitreous correlate with the extent and duration of rhegmatogenous retinal detachment [5,6]. The results from these reports suggest that increased vitreous MMP-9 may play a role in conditions where the RPE-neurosensory retina interface has been compromised. This reaction to increased MMP-9 expression is logical because as membranes and collagen are being broken down, areas are left open for fluid accumulation and new blood vessel growth. Currently, we can only speculate what role MMP-9 might play in forming SRF. Since what causes the development of SRF is not yet understood, an array of proteins might be involved in the normal attachment of the outer retina segments to the RPE.

Our study builds on past findings by showing that levels of MMP-9 in the vitreous of patients with AMD are significantly associated with the accumulation of SRF. These results also help further define the biologic mechanisms involved in the disease. Although each sample used in this study was screened against 37 antibodies to growth factors, cytokines, and other proteins, MMP-9 had the most significant findings in this cohort of patients (see Table 4). Between the two groups of patients, the mean vitreous levels of MMP-9 were statistically higher in patients with SRF (p<0.0001). In addition, we observed a positive correlation between vitreous levels of MMP-9 and the height of SRF, suggesting that a direct relationship between disease activity and vitreous MMP-9 may be present (see Figure 4). No such correlation with CRT was observed. Data shown in Figure 4 shows that elevation of MMP-9 preceded elevation of SRF. This suggests that MMP-9 levels may have some prognostic significance clinically. The patients in Group 1 with higher vitreous MMP-9 expression levels and higher SRF exhibited more persistent disease compared with patients without SRF (Figure 3). In Group 1, only one patient (Patient 1) had full resolution of SRF, while all others continued to have elevated SRF levels. Vitreous levels of MMP-9 might be able to predict levels of SRF as is suggested by the results from the ROC curve analysis (see Figure 5). To validate this claim, a true prediction model will need to be developed as more data are collected.

With regard to retinal thickening, an additional finding was observed in Group 1. In this group there was minimal retinal thickening overlying areas of SRF. In addition, retinal thickness remained relatively constant despite significant changes in SRF levels (Appendix 1). These observations combined with the vitreous MMP-9 data may suggest an alternative biologic pathway for retinal edema and SRF development that is perhaps partly mediated by MMP-9 activity. Future studies of other retinal conditions such as central serous retinopathy will determine if there is also a correlation between MMP-9 and SRF.

A limitation of the current study is the small patient population size. However, we analyzed 62 total samples with three to nine samples per eye during the course of disease. To the best of our knowledge, this is the largest group yet reported of in-office vitreous samples at multiple time points during a treatment course. Previous studies used surgical samples; however, one is unable to track a patient’s response over multiple time points using vitrectomy samples as a guide. The advantage of this study was the ability to monitor individual patients’ proteomic response at multiple time points while being treated for wet AMD with intravitreal therapies. We hope that in the future as more research is completed, in-office vitreous diagnostics will enable the physician to better understand disease and predict future clinical course. Surely, this would be a useful advancement toward individualized treatment plans.

### Conclusion

This pilot study has shown that in a small subset of patients with different morphologic forms of exudative AMD (SRF, CRT), patients with subretinal fluid have significantly higher vitreous levels of MMP-9 versus those with only retinal thickening. A positive correlation with the vitreous level of MMP-9 and the level of subretinal fluid was observed and shown. Larger studies including more patients who have had DVS sampling will be helpful to further assess the relationship between MMP-9 and other vitreous proteins with SRF levels in patients with wet AMD and other neovascular retinal diseases.

## Appendix 1. Patient Sample Intervals and Clinical Outcome.

To access these data, click or select the words “Appendix 1.” This will initiate the download of a compressed (zip) archive that contains the pdf file.

## Appendix 2. List of Antibodies and Manufactures Used in this Study.

To access these data, click or select the words “Appendix 2.” This will initiate the download of a compressed (zip) archive that contains the pdf file.

## References

[1] Davuluri G, Espina V, Petricoin EF, Ross M, Deng J, Liotta LA, Glaser BM (2009). Activated VEGF Receptor Shed into the Vitreous in Eyes with Wet AMD.. Arch Ophthalmol.

[2] Guymer RH, Tao LW, Goh JK, Liew D, Ischenko O, Robman LD, Aung K, Cipriani T, Cain M, Richardson AJ, Baird PN, Langham R (2011). Identification of Urinary Biomarkers for Age-Related Macular Degeneration.. Invest Ophthalmol Vis Sci.

[3] Seddon JM, George S, Rosner B, Rifai N (2005). Progression of Age-Related Macular Degeneration Prospective Assessment of C-Reactive Protein, Interleukin 6, and Other Cardiovascular Biomarkers.. Arch Ophthalmol.

[4] Pfahler SM, Brandford A, Glaser BM (2009). A Prospective Study of In-Office Diagnostic Vitreous Sampling in Patients with Vitreo-Retinal Pathology.. Retina.

[5] Symeonidis C, Diza E, Papakonstantinou E, Souliou E, Karakiulakis G, Dimitrakos SA (2007). Expression of Matrix Metalloproteinases in the Subretinal Fluid Correlates with the extent of Rhegmatogenous Retinal Detachment.. Graefes Arch Clin Exp Ophthalmol.

[6] Symeonidis C, Diza E, Papakonstantinou E, Souliou E, Dimitrakos SA, Karakiulakis G (2007). Correlation of the Extent and Duration of Rhegmatogenous Retinal Detachment with the Expression of Matrix Metalloproteinase in the Vitreous.. Retina.

[7] Espina V, Mehta AI, Winters ME, Calvert V, Wulfkuhle J, Petricoin EF, Liotta LA (2003). Protein Microarrays: Molecular Profiling Technologies for Clinical Specimens.. Proteomics.

[8] Boja ES, Rodriguez H (2011). The Path to Clinical Proteomics Research: Integration of Proteomics, Genomics, Clinical Laboratory and Regulatory Science.. Korean J Lab Med.

[9] Steen B, Sejersen S, Berglin L, Seregard S, Kvanta A (1998). Matrix Metalloproteinases and Metalloproteinase Inhibitors in Choroidal Neovascular Membranes.. Invest Ophthalmol Vis Sci.

[10] Lommatzsch A, Hermans P, Müller KD, Bornfeld N, Bird AC, Pauleikhoff D (2008). Are Low Inflammatory Reactions involved in exudative age-related macular degeneration?. Graefes Arch Clin Exp Ophthalmol.

[11] Hoffman S, He S, Ehren M, Ryan SJ, Wiedemann P (2006). MMP-2 and MMP-9 Secretion by RPE is stimulated by angiogenic molecules found in Choroidal Neovascular Membranes.. Retina.

[12] Kvanta A, Shen W, Sarman S, Seregard S, Steen B, Rakoczy E (2000). Matrix Metalloproteinase (MMP) expression in experimental choroidal neovascularization.. Curr Eye Res.

[13] Lambert V, Munaut C, Jost M, Noël A, Werb Z, Foidart J, Rakic AJ-M (2002). Matrix Metalloproteinase-9 Contributes to Choroidal Neovascularization.. Am J Pathol.

[14] Chau KY, Sivasprasad S, Patel N (2008). Plasma levels of Matrix Metalloproteinase-2 and −9 (MMP-2 and MMP-9) in Age-Related Macular Degeneration.. Eye (Lond).

[15] Plantner JJ, Smine A, Quinn TA (1998). Matrix Metalloproteinases and Metalloproteinase Inhibitors in Human Interphotoreceptor matrix and Vitreous.. Curr Eye Res.

